# Patient-Derived Xenograft Models for Intrahepatic Cholangiocarcinoma and Their Application in Guiding Personalized Medicine

**DOI:** 10.3389/fonc.2021.704042

**Published:** 2021-07-13

**Authors:** Yang Gao, Rong Zhou, Jun-Feng Huang, Bo Hu, Jian-Wen Cheng, Xiao-Wu Huang, Peng-Xiang Wang, Hai-Xiang Peng, Wei Guo, Jian Zhou, Jia Fan, Xin-Rong Yang

**Affiliations:** ^1^ Department of Liver Surgery & Transplantation, Liver Cancer Institute, Zhongshan Hospital, Fudan University, Key Laboratory of Carcinogenesis and Cancer Invasion, Ministry of Education, Shanghai, China; ^2^ Department of Blood Transfusion, Zhongshan Hospital, Fudan University, Shanghai, China; ^3^ Department of Intensive Care Medicine, Zhongshan Hospital, Fudan University, Shanghai, China; ^4^ Shanghai Dunwill Medical Technology Co., Ltd., Shanghai, China; ^5^ Shanghai Epione Medlab Co., Ltd., Shanghai, China; ^6^ Department of Laboratory Medicine, Zhongshan Hospital, Fudan University, Shanghai, China; ^7^ Institutes of Biomedical Sciences, Fudan University, Shanghai, China

**Keywords:** patient derived xenograft, intrahepatic cholangiocarcinoma, lenvatinib, drug resistance, personalized medicine

## Abstract

**Background:**

Intrahepatic cholangiocarcinoma (ICC) remains one of the most intractable malignancies. The development of effective drug treatments for ICC is seriously hampered by the lack of reliable tumor models. At present, patient derived xenograft (PDX) models prove to accurately reflect the genetic and biological diversity required to decipher tumor biology and therapeutic vulnerabilities. This study was designed to investigate the establishment and potential application of PDX models for guiding personalized medicine and identifying potential biomarker for lenvatinib resistance.

**Methods:**

We generated PDX models from 89 patients with ICC and compared the morphological and molecular similarities of parental tumors and passaged PDXs. The clinicopathologic features affecting PDX engraftment and the prognostic significance of PDX engraftment were analyzed. Drug treatment responses were analyzed in IMF-138, IMF-114 PDX models and corresponding patients. Finally, lenvatinib treatment response was examined in PDX models and potential drug resistance mechanism was revealed.

**Results:**

Forty-nine PDX models were established (take rate: 55.1%). Successful PDX engraftment was associated with negative HbsAg (P = 0.031), presence of mVI (P = 0.001), poorer tumor differentiation (P = 0.023), multiple tumor number (P = 0.003), presence of lymph node metastasis (P = 0.001), and later TNM stage (P = 0.039). Moreover, patients with tumor engraftment had significantly shorter time to recurrence (TTR) (P < 0.001) and worse overall survival (OS) (P < 0.001). Multivariate analysis indicated that PDX engraftment was an independent risk factor for shortened TTR (HR = 1.84; 95% CI, 1.05–3.23; P = 0.034) and OS (HR = 2.13; 95% CI, 1.11–4.11; P = 0.024). PDXs were histologically and genetically similar to their parental tumors. We also applied IMF-138 and IMF-114 PDX drug testing results to guide clinical treatment for patients with ICC and found similar treatment responses. PDX models also facilitated personalized medicine for patients with ICC based on drug screening results using whole exome sequencing data. Additionally, PDX models reflected the heterogeneous sensitivity to lenvatinib treatment and CDH1 might be vital to lenvatinib-resistance.

**Conclusion:**

PDX models provide a powerful platform for preclinical drug discovery, and potentially facilitate the implementation of personalized medicine and improvement of survival of ICC cancer patient.

## Introduction

Intrahepatic cholangiocarcinoma (ICC) is the second most common primary liver tumor, accounting for approximately 10%-15% of primary hepatic malignancies (1, 2). Moreover, the overall incidence of ICC shows a significant increasing trend worldwide with an annual percentage increase of 2.30% in the past four decades (3, 4). Surgical resection remains the only potentially curative option for patients with ICC, but less than 25% of patients with ICC are eligible for resection (5). Most patients (approximately 70%) are diagnosed at late stages and systematic therapies are recommended (5), while the lack of effective treatment regimens results in dismal outcomes for those patients (6). Despite considerable advances in understanding ICC complexity of this kind of tumor, the development of an effective antitumor drug for ICC patients hasn’t progressed due to a lack of suitable preclinical models that recapitulate the pathologic, biological, and genetic features of ICC.

At present, the use of patient derived xenograft (PDX) models has become an attractive platform for drug development and translational cancer research (7, 8). PDX models are established by directly engrafting fresh tumor tissue during surgery or biopsy into immunodeficient mice, which can recapitulate human tumor biology more accurately than traditional cell line-derived xenografts (9). Cancer organoid is an *in vitro* 3D culture model from patient tumor and we also some liver cancer organoid model, which potentially enables drug screening and individualized treatment regimen and was regarded as an intermediate modeling platform in precision oncology (10–12). PDX is a kind of *in vivo* tumor model and has unique advantage in basic research and translational medicine. More importantly, the consistency of treatment responses between PDX models and corresponding patients has been confirmed in several types of solid tumors, including our recent research (13–16). Therefore, PDX model provides an alternative preclinical model for the discovery of novel treatment regimens for patients with malignant tumors. Recently, Wang et al. constructed a cholangiocarcinoma PDX biobank with an engraftment rate of 83.3% (30 of 36) and used these models to reveal the chemosensitivity of proteasome inhibition in cholangiocarcinoma, which further broadened the application in medical research (17). The first report on ICC PDX model was published in 2016 by Giuliana et al., who successfully established and confirmed the histological and genetic similarity between primary tumor and PDX model (18). However, the tumor take rate was extremely low, and only one out of 17 tumors (5.8%) was successfully engrafted (18). Furthermore, the clinical significance of PDX engraftment and its potential applications in guiding personalized medicine still need to be further explored.

Here, we report the establishment of a unique bank of serially transplantable tumor grafts that retain crucial characteristics of the original tumor specimens from ICC patients based on our previous independently developed technique for PDX model engraftment in HCC (15). We found that successful PDX engraftment was significantly associated with presence of microvascular invasion (mVI), poorer tumor differentiation, multiple tumor number, presence of lymph node metastasis, and advanced tumor stage in patients with ICC. In addition, successful tumor engraftment indicated shorter time to recurrence (TTR), and overall survival (OS). This PDX model shows great promise in a preclinical setting for biomarker development, and will aid in understanding the mechanisms of drug resistance, drug screening as well as personalized medicine applications for patients with ICC.

## Materials and Methods

### Patients and Tumor Samples

From June 2014 to December 2019, 89 patients with ICC receiving treatment in Zhongshan Hospital of Fudan University were included in this study. ICC was defined based on pathologic diagnosis from formalin-fixed, paraffin-embedded tissue or biopsy tissue. All patients received curative resection (defined as complete excision with negative tumor margins) (19). Tumor staging was based on the Seventh American Joint Committee on Cancer (AJCC) TNM staging system (20). This study was approved by the Ethics Board of the Liver Cancer Institute of Zhongshan Hospital, Fudan University (Shanghai, China) and informed written consent was obtained from each patient. TTR was defined as the interval between the date of surgery and the date of clinical diagnose of tumor recurrence. OS was defined as the interval between the date of surgery and the date of death from any cause or the last follow-up visit. Follow up was terminated on September 1, 2020.

### Establishment of Xenografts

All mouse experiments were approved by the Animal Care and Use Committee of Zhongshan Hospital of Fudan University. Three-week-old male, nonobese diabetic/severe combined immunodeficient (NOD/SCID) or NOG (NOD/Shi-scid, IL-2Rγnull) mice were used for tumor engraftment. Following surgery, tumor samples were collected in serum-free DMEM media (Dulbecco’s Modified Eagle’s Medium, Gibco, United States) supplied with 100 U/ml penicillin and 100 μg/ml streptomycin (Gibco, United States) for engraftment within 1 hour after surgery. Tumor samples (2×4×4 mm^3^) were subcutaneously implanted into the right flank of each mouse. Tumor growth was measured every 3 days using calipers. To verify the establishment of PDX in each mouse, the tumors were monitored for at least 3 months. When the tumor volumes reached 1000 mm^3^, the mice were sacrificed and tumors were excised into small fragments and implanted into another cohort of mice, frozen for morphological, genetic expression analysis or whole exome sequencing.

### Hematoxylin and Eosin Staining and Immunohistochemistry

Patient tumor specimens and PDX tissues were formalin fixed, paraffin-embedded, cut into sections (4 μm thick), and stained with hematoxylin and eosin (H&E). The biliary cancer marker cytokeratin (CK) 7 and CK19 (21), endothelial marker CD34 (22) and nuclear proliferation biomarker Ki67 (23) were evaluated by immunohistochemical analysis (IHC) to compare the morphological similarity among tumor tissues and passaged PDXs. Primary antibodies CK7 (1:200), CK19 (1:300), CD34 (1:100), and Ki67 (1:200) were purchased from Abcam (Canbridge, MA, USA). Immunohistochemistry (IHC) was performed using a two-step protocol as previously described (24). Pathological examinations were performed under light microscopy by two pathologists blinded to the clinical information of patients.

### Whole Exome Sequencing

Genomic DNA were extracted from tumor tissues by means of the Allprep DNA FFPE extraction kit (Qiagen, Germantown, MD), according to the manufacturer’s instructions. After purification, DNA was eluted in 30 µL of water, and yield was determined by the Qubit DNA HS Assay (from Thermo Fisher Scientific, Waltham, MA), according to the manufacturer’s recommendation. A predefined yield of 300 ng of DNA was used as acceptance criteria to ensure adequate library preparation. DNA-seq libraries were constructed using the TruSeq DNA exome Kit (Illumina, Inc., San Diego, CA, United States) following the manufacturer’s recommendations. After library quality and quantity assessment, DNA-seq samples were sequenced using an Illumina HiSeq x10 instrument (pair-end 150 cycle reactions). First, sequencing reads were mapped/aligned to reference genome hg19 using the Burrows-Wheeler Aligner (BWA). Second, mapped/aligned reads were sorted and indexed with Samtools 0.1.19. Third, Picard-tools were used to fix mate pair and remove duplicate reads. Fourth, INDEL realignment was conducted with the Genome Analysis Toolkit (GATK 4.1.1). Fifth, base quality was recalibrated by GATK using dbSNP151. Then, GATK Mutect2 was used to detect SNP/Indels mutations with tumor-normal paired mode. During this step, gnomAD v2.1.1 was used to filter germline mutations. False positive mutations were filtered using GATK FilterMutectCalls, and only “PASS”-labeled mutations remained. The functional effect of variants called were annotated by ANNOVAR (25).

### *In Vivo* Pharmacologic Studies

Passage 3– 4 PDX models were used to evaluate the efficiency of various treatment regimens for ICC. When the tumor volumes reached approximately 100–150 mm^3^, mice were randomly divided into groups with 5 mice/group. Groups were treated as follows: (1) control group received weekly intraperitoneal (i.p.) injection of 100 μl phosphate buffered saline; (2) GEMOX group received gemcitabine (50 mg/kg, i.p. once per week) + oxaliplatin (5 mg/kg, i.p. once per week); (3) XELOX group received capecitabine (200 mg/kg, orally, once per day) + oxaliplatin (5 mg/kg, i.p. once per week); (4) FOLFOX group received oxaliplatin (5 mg/kg, i.p. once per week) + 5-FU (25 mg/kg, i.p. once per week); (5) sorafenib group received sorafenib (30 mg/kg orally, once per day); (6) lenvatinib group received lenvatinib (30 mg/kg, orally, once per day); (7) alpelisib group received alpelisib (50 mg/kg, orally, once per day); (8) abemaciclib group received abemaciclib (50 mg/kg, orally, once per day); (9) copanisib received copanisib (6 mg/kg, orally, once per day); and (10) trametinib received trametinib (1 mg/kg, orally, once per day). All drugs were purchased from MedChemExpress Co., Ltd. (Monmouth Junction, NJ, United States). Animals were treated for 4 weeks. All animals were weighed at the time of tumor measurement and were observed daily for physical appearance, behavior, and clinical changes. Tumor volumes were calculated using the formula: V = ½ × length × (width)^2^, where length = the greatest longitudinal diameter and width = the greatest transverse diameter. Tumor growth inhibition (TGI) was calculated by relative treated group tumor growth (ΔT) divided by relative control group tumor growth (ΔC), which can be expressed by: TGI = ΔT/ΔC (26). We adopted the Division of Cancer Treatment of the National Cancer Institute (NCI) criteria in this study and defined tumor response as 0%–20% TGI, tumor stability as 21%–50% TGI, and tumor progression > 50% TGI (27).

### Statistical Analysis

Statistical analysis was performed using SPSS 19.0 software (IBM SPSS Inc., Chicago, IL, USA). χ^2^ and Fisher’s exact probability tests were used to compare the association between clinicopathological factors and engraftment. Survival analyses were performed using the Kaplan–Meier method and survival curves were compared by log-rank test. Variables of interest were tested using Cox proportional hazards regression analysis and significant variables obtained identified by univariate analysis were subjected to multivariate analysis (28). All statistical analyses were two-sided and a P-value < 0.05 was considered statistically significant.

## Results

### The Patient Characteristics and Establishment of PDX Models

Patient characteristics are shown in **Table 1**. The patient cohort included 43 men and 46 women with a median age of 59 years (range, 34–85 years). Finally, 49 of the 89 engrafted tumors were successfully established as PDX models (take rate: 55.1%). We compared clinical characteristics and successful PDX establishment and found that the PDX engraftment rate was associated with negative HbsAg (P = 0.031), presence of mVI (P = 0.001), poorer tumor differentiation (P = 0.023), multiple tumor number (P = 0.003), presence of lymph node metastasis (P=0.001), and later TNM stage (P = 0.039) (**Table 1**).

**Table 1:** The relationship between clinicopathological factors and PDX take rates.

Variations	Number of patientsN = 89	Establishment of PDX		P value
		No (n = 40)	Yes (n = 49)	
Sex				0.572
Male	43	18	25	
Female	46	22	24	
Age (years)				0.311
≤50	18	10	8	
>50	71	30	41	
GGT (U/ml)				0.386
≤54	40	20	20	
>54	49	20	29	
HbsAg				**0.031**
Negative	49	17	32	
Positive	40	23	17	
CA19-9 (U/ml)				0.973
≤37	38	17	21	
>37	51	23	28	
Tumor size (cm)				0.103
≤5	34	19	15	
>5	55	21	34	
Capsule formation				0.310
No	79	34	45	
Yes	10	6	4	
mVI				**0.001**
Absence	55	32	23	
Presence	34	8	26	
Tumor differentiation				**0.023**
I-II	33	20	13	
III-IV	56	20	36	
Tumor number				**0.003**
Solitary	61	34	27	
Multiple	28	6	22	
Lymph node metastasis				**0.001**
Absence	65	36	29	
Presence	24	4	20	
TNM stage				**0.039**
I-II	54	29	25	
III-IV	35	10	25	
Liver cirrhosis				0.889
No	46	21	25	
Yes	43	19	24	

GGT, Glutamyl transpeptidase; HBsAg, hepatitis B surface antigen; CA19-9, carbohydrate antigen 19-9; mVI, microvascular invasion; TNM, tumor-node-metastasis; PDX, patient derived xenograft; NA, not applicable.

Statistically significant (P < 0.05) values are in bold.

### The PDX Models Recapitulated Primary Tumors in the Histologic, Molecular and Genomic Levels

**Figure 1:**
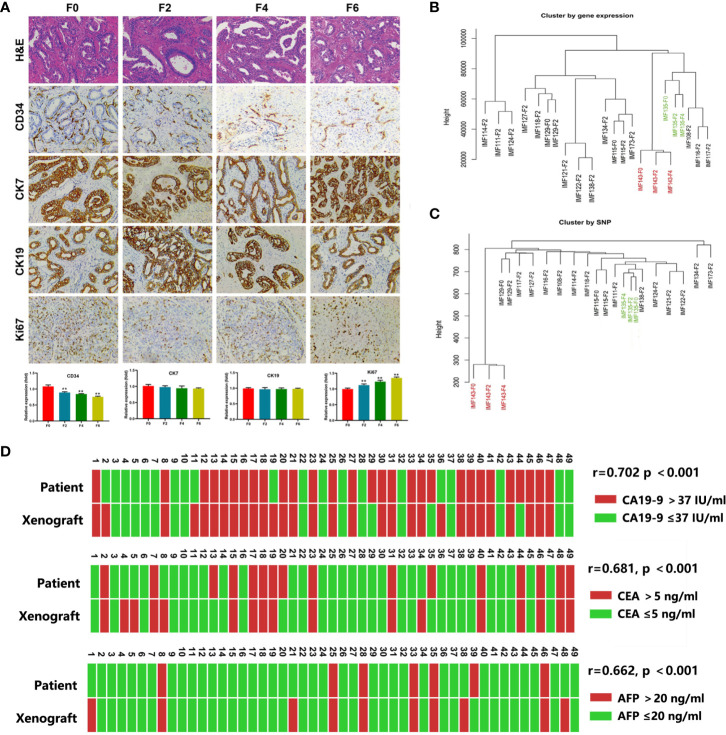
The PDX models recapitulated the characteristics of the parental tumors. **(A)** Representative H&E sections and immunohistochemical profiles of CD34, CK7, CK19 and Ki67 in serial PDXs and their parental primary tumors (200×). F0, primary tumor; F2, the second passaged xenograft in mice; F4 and F6, the fourth and sixth passaged xenograft; **P < 0.01 **(B, C)**, The dendograms showed unsupervised clustering of samples according to gene expression pattern by Human Genome U133 Plus 2.0 Array **(B)** and SNP by SNP 6.0 arrays **(C)**; **(D)** Comparison of serum CA19-9, CEA and AFP in PDX models and corresponding patient.

The histologic, molecular, and genomic relevance of the engrafted tumors were compared with those of the primary tumors (between F0, F2, F4, and F6). F0 indicated primary tumor and F2, F4 and F6 indicated the second, fourth and sixth passaged xenograft in mice The morphological features of serial passages, as shown by H&E-staining, were well maintained, and tumors of PDX models resembled the original tumors at the cellular and structural levels. The molecular features of the tumors were assessed by immunohistochemical analysis of currently used diagnostic markers. CK7 and CK19 expression in PDX models were mostly consistent with that in the parental primary tumors. In contrast, Ki67 expression was weak in the earlier PDX passages and became stronger in the later PDX passages. CD34 expression also gradually decreased, implying that human stromal elements are replaced by murine stroma as the tumor was engrafted in the new microenvironment (**Figure 1A**).

We next evaluated the maintenance of gene expression and single nucleotide polymorphism (SNP) using Human Genome U133 Plus 2.0 Array and SNP 6.0 arrays, respectively and some pairs of parental primary tumors and PDX models (F2–F4 xenograft tumors) were compared. Unsupervised clustering of gene expression and SNP analysis showed that all the PDXs could be tightly clustered with their corresponding primary tumors (**Figures 1B, C**).

CA19-9 is routinely used in ICC diagnosis and follow-up. Therefore, we also explored the relationship between CA19-9 levels in PDX models and corresponding patients. We found elevated CA19-9 in 60.4% (29/48) of PDXs (median, 86.3 U/ml; range, 0.6 to > 10,000 U/ml), which was consist with corresponding levels in patients (median, 51.7 U/ml; range, 0.6 to > 10,000 U/ml; r = 0.702, p < 0.001). Similar AFP and CEA levels were observed in PDXs and corresponding patients (**Figure 1D**).

### The Prognostic Value of Tumor Engraftment in ICC

**Figure 2:**
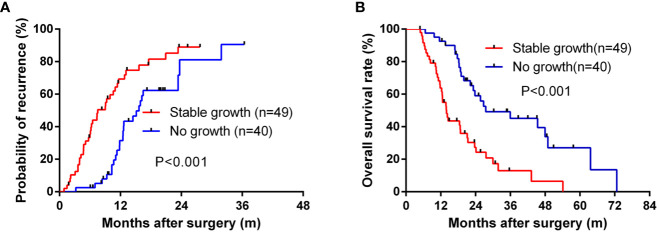
The Kaplan–Meier analysis of TTR and OS for the stable growth of grafts. **(A)** for TTR and **(B)** for OS.

To determine the influence of engraftment ability on clinical outcomes, we assessed TTR and OS in patients with tumors used to create PDX models. Tumor engraftment was significantly associated with shorter TTR (median, 7.53 months versus 15.67 months; P < 0.001; **Figure 2A**) and OS (median, 13.97 versus 27.50 months; P < 0.001; **Figure 2B**). Furthermore, univariate analysis revealed that the ability to engraft was a prognostic factor for TTR (HR = 2.36; 95% CI, 1.40–3.98; P = 0.001) and OS (HR = 2.65; 95% CI, 1.48–4.72; P = 0.001) (**Table 2**). Multivariate analysis was conducted and the ability to generate a stable engraftment was an independent prognostic factor for TTR (HR = 1.84; 95% CI, 1.05–3.23; P = 0.034) and OS (HR = 2.13; 95% CI, 1.11–4.11; P = 0.024).

**Table 2:** Univariate and multivariate analyses showed PDX establishment as an independent predictor for TTR and OS.

Variables	TTR		OS	
	HR (95% CI)	P value	HR (95% CI)	P value
**Univariate analyses**				
Sex (Female *vs* male)	0.98 (0.59-1.61)	0.924	1.21 (0.70-2.09)	0.504
Age, years (>50 *vs* ≤ 50)	1.63 (0.83-3.19)	0.153	1.55 (0.73-3.30)	0.256
GGT, U/ml (>54 *vs* ≤54)	1.94 (1.15-3.28)	**0.013**	2.13 (1.20-3.76)	**0.009**
HBsAg (Positive *vs* negative)	0.96 (0.58-1.58)	0.955	0.94 (0.55-1.63)	0.839
CA19-9,U/ml (>37 *vs* ≤37)	1.55 (0.92-2.62)	0.099	1.88 (1.05-3.35)	**0.034**
Tumor size, cm (>5 *vs* ≤5)	1.26 (0.74-2.16)	0.393	1.79 (0.99-3.22)	0.052
Capsule formation (Yes *vs* no)	0.82 (0.37-1.81)	0.620	0.78 (0.33-1.84)	0.572
mVI (Presence *vs* absence)	1.61 (0.97-2.67)	0.065	1.84 (1.05-3.20)	**0.032**
Tumor differentiation (III-IV *vs* I-II)	1.19 (0.70-2.04)	0.520	1.13 (0.64-2.00)	0.676
Tumor number (Multiple *vs* solidary)	2.13 (1.27-3.58)	**0.004**	2.20 (1.26-3.85)	**0.006**
TNM Stage (III-IV *vs* I-II)	1.71 (1.02-2.85)	**0.042**	1.82 (1.04-3.19)	**0.035**
Liver cirrhosis (Yes *vs.* no)	0.90 (0.54-1.47)	0.889	1.12 (0.65-1.95)	0.678
PDX establishment (Yes *vs* no)	2.36 (1.40-3.98)	**0.001**	2.65 (1.48-4.72)	**0.001**
**Multivariate analyses**				
GGT, U/ml (>54 *vs* ≤54)	1.85 (1.08-3.15)	**0.024**	1.85 (1.01-3.38)	**0.047**
CA19-9,U/ml (>37 *vs* ≤37)	NA	NA	1.68 (0.91-3.08)	0.097
mVI (Presence *vs* absence)	NA	NA	1.07 (0.57-2.02)	0.832
Tumor number (Multiple *vs* solidary)	1.60 (0.91-2.80)	0.103	1.46 (0.77-2.74)	0.245
TNM Stage (III-IV *vs* I-II)	1.62 (0.96-2.73)	0.073	1.80 (1.01-3.22)	**0.046**
PDX establishment (yes *vs* No)	1.84 (1.05-3.23)	**0.034**	2.13 (1.11-4.11)	**0.024**

TTR, time to recurrence; OS, Overall survival; GGT, Glutamyl transpeptidase; HBsAg, hepatitis B surface antigen; CA19-9, carbohydrate antigen 19-9; mVI, microvascular invasion; TNM, tumor-node-metastasis; PDX, patient derived xenograft; NA, not applicable; HR, hazard ratio; CI, confidence interval.

Statistically significant (P < 0.05) values are in bold.

### The Application of PDX Model in Clinical Individualized Treatment for Patients With ICC

**Figure 3:**
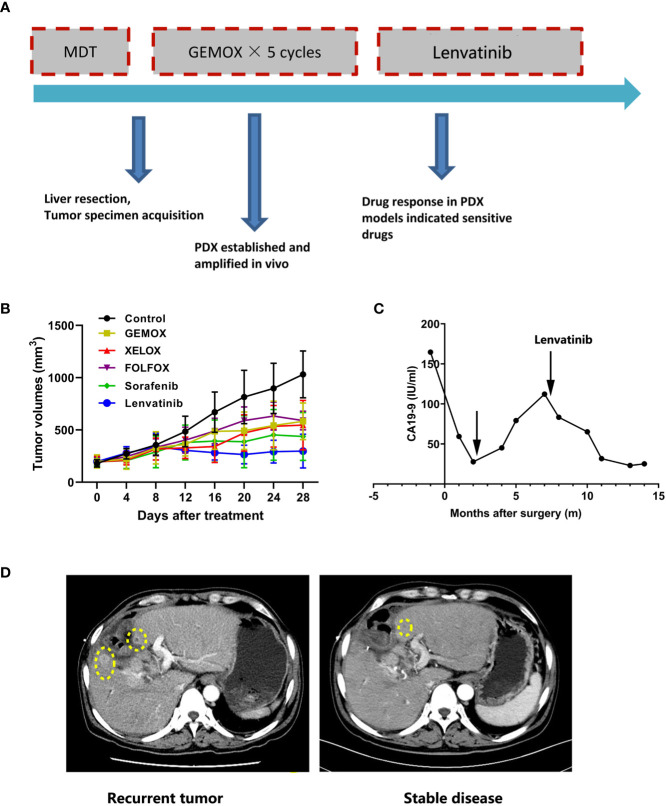
The PDX model guide personalized treatment for ICC patients. **(A)** Summary of clinical history for IMF-138; **(B)** PDX models showed diverse treatment response to various treatment regimens; **(C)** Human CA19-9 levels were observed during the clinical course; **(D)** The representative CT images for patient who received lenvatinib treatment.

To further demonstrate the value of the PDX model in clinical individualized treatment for patients with ICC, two typical ICC patients with established corresponding PDX models were assessed. Patient IMF-138 was initially diagnosed with hepatic lesion and received curative liver resection plus lymphadenectomy. Pathological and immunohistological findings confirmed a moderately differentiated adenocarcinoma, leading to a final diagnose of ICC (pT3N0M0 pStage III)s. After the operation, the patient received adjuvant systemic treatment (GEMOX regimen). However, the patient suffered tumor recurrence during GEMOX treatment (**Figure 3A**). In parallel, we tested the PDX model with some commonly used regimens (GEMOX, XELOX, FOLFOX, sorafenib, and lenvatinib) and found that the PDX model showed much better response to lenvatinib than other regimens. Consistent with the observed treatment response in patient IMF-138 in clinical practice, the model was not very sensitive to GEMOX regimen **(** **Figure 3B**). CA19-9 level was assessed as a surrogate marker of ICC, and was observed to be increased when tumor recurrence occurred (**Figure 3C**). Based on PDX model drug sensitivity results, lenvatinib was administered to the patient and resulted in tumor shrinkage and stable disease until the last visit was achieved (**Figure 3D**).

**Figure 4:**
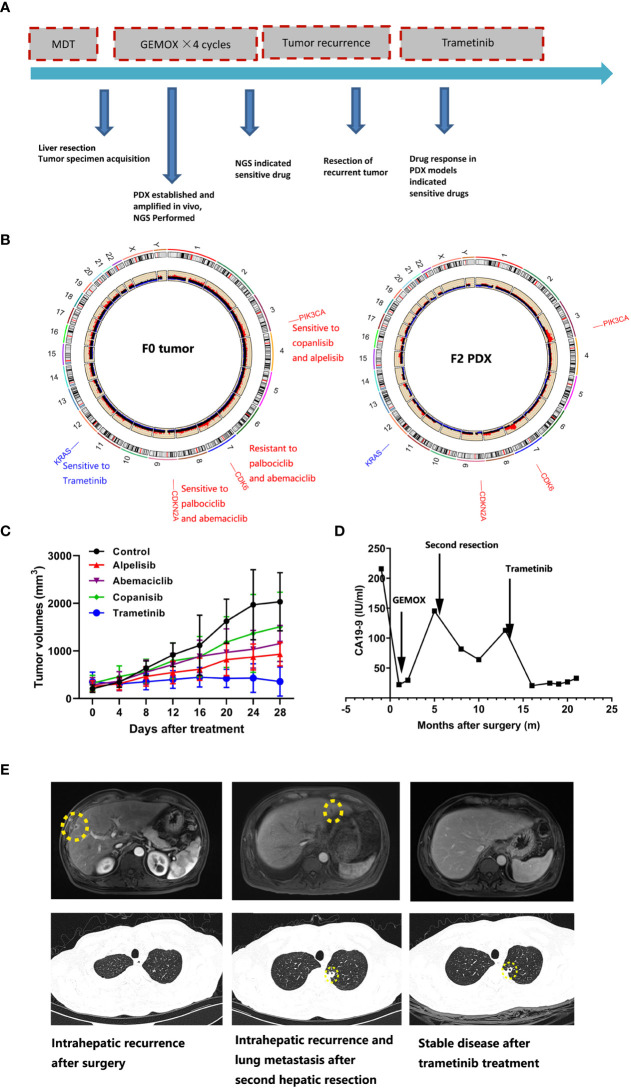
The PDX model combined with the whole exome sequencing data guide drug selection for ICC patients. **(A)** Summary of clinical history for IMF-11; **(B)** Whole exome sequencing identified gene mutations with potential clinical value; right figure indicated F0 tumor and left figure indicated F2 xenograft tumor; **(C)** PDX models showed diverse treatment response to various treatment regimens; **(D)** Human CA19-9 levels were observed during the clinical course; **(E)** The representative hepatic MRI and lung CT images for patient who received trametinib treatment.

Patient IMF-114 was initially diagnosed with malignant liver tumor and received partial liver resection plus lymphadenectomy. Pathological examination showed a poorly differentiated adenocarcinoma, leading to a final diagnose of ICC (pT3N1M1 pStage IV) (**** **Figure 4A**). The tumor tissues were subjected to whole exome sequencing (WES) and four mutations with potential clinical significance were identified: CDKN2A deletion, CDK6 amplification, PIK3CA amplification, and KRAS G12V mutation (**** **Figure 4B**). Additionally, PDX models (F2 xenograft tumor) showed a similar mutation landscape to that of the F0 tumor, further validating that the PDX model recapitulated the genomic characteristics of the original tumor. After construction of the matched PDX model, we used it to test treatment responses to the potential drugs based on WES. We found that trametinib produced the best TGI in the PDX model (**Figure 4C**). During follow-up, 8 months after surgery, patient IMF-114 developed intrahepatic recurrence and lung metastasis during adjuvant GEMOX regimen administration (**Figures 4D, E** **)**. Based on the drug test results, we decided to try treatment with trametinib in this patient. After trametinib administration, intrahepatic metastasis was suppressed, lung metastasis was stable for 7 months, and CA19-9 decreased to normal levels (**Figures 4D, E** **)**. At the time of this manuscript preparation the patient is still alive and has survived for 22 months from the date of surgery.

### Applying ICC PDX Models to Reveal Potential Lenvatinib Resistance Mechanism

**Figure 5:**
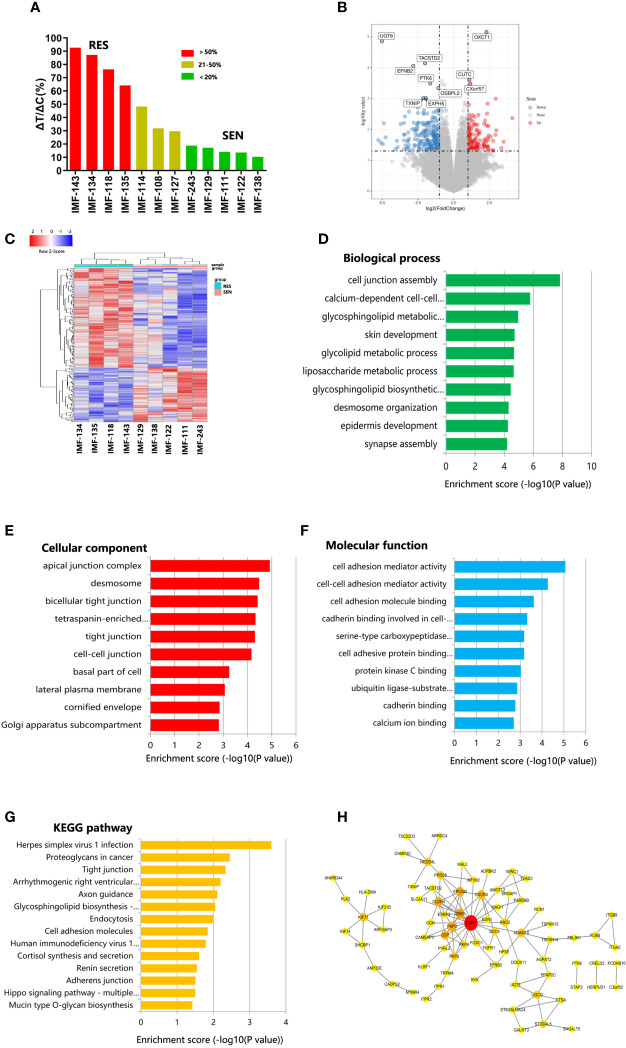
PDX models revealed potential mechanism of lenvatinib resistance. **(A)** Waterfall plot of lenvatinib response after 4 weeks of treatment in 12 cases. Resistant, stable and sensitive cases are shaded in red, yellow and green, respectively. RES, resistant; SEN, sensitive; **(B)** Volcano plot represents significantly differentially expressed genes in lenvatinib-sensitive group and lenvatinib-resistant. Selected top up- and downregulated genes are labeled; **(C)** Heat map of differentially expressed gene between lenvatinib-sensitive and lenvatinib-resistant PDXs. **(D–F)** GO analysis of differentially expressed gene according to biological process, cellular component and molecular function, respectively; **(G)** Pathway analysis based on the KEGG database; **(H)** Protein–protein interaction (PPI) networks for differentially expressed proteins. The gene network was constructed using Cytoscape software based on the STRING database.

We explored the mechanism of lenvatinib drug resistance in ICC, by testing 12 established PDX models with lenvatinib (30 mg/kg/day) for 28 days. The PDX models reflected diverse treatment responses to lenvatinib administration. Five PDX models (IMF-138, IMF-122, IMF-111, IMF-129, and IMF-243) were sensitive to lenvatinib treatment, while another four PDX models (IMF-143, IMF-134, IMF-118, and IMF-135) were resistant to the same treatment regimen (**Figure 5A**). These results indicate that the PDX models reflect the heterogeneity of lenvatinib response observed in patients with ICC.

To reveal the potential mechanism of lenvatinib resistance in ICC, we compared SNP and gene expression in five lenvatinib-sensitive PDXs and four lenvatinib-resistant PDXs. Using a P-value < 0.05 and fold change > 2 to compare lenvatinib-sensitive PDXs lenvatinib-resistant PDXs, we identified 64 and 117 genes that were up- and down-regulated (**Figures 5B, C**). Differentially expressed genes were subjected to gene ontology (GO) and Kyoto encyclopedia of genes and genomes (KEGG) analyses. GO analysis provides information about biological processes, cellular components, and molecular function (**Figures 5D–F**). With respect to molecular functions, the most highly enriched GO terms in the differentially expressed transcripts included cell adhesion mediator activity, cell–cell adhesion mediator activity, cell adhesion molecule binding, cadherin binding involved in cell–cell adhesion, and serine-type carboxypeptidase activity. Biological processes were mainly enriched in cell junction assembly, calcium-dependent cell–cell adhesion *via* plasma membrane cell adhesion molecules, glycosphingolipid metabolic process, skin development, and glycolipid metabolic process. GO cellular components show that the differentially expressed genes were significantly located in the apical junction complex, desmosome, bicellular tight junction, tetraspanin-enriched microdomain, and tight junctions. KEGG pathway analysis of differentially expressed genes showed that they were enriched in proteoglycans in cancer, tight junctions, cell adhesion molecules, and the Hippo signaling pathway, which may harbor significance in mediating lenvatinib resistance in ICC (**Figure 5G**).

To better understand the role of the differentially expressed genes in lenvatinib resistance, protein–protein interaction (PPI) analysis was also performed. All DEGs were submitted to STRING 11.0 (29), and we obtained 77 PPI nodes and 117 edges. Finally, PPI analysis revealed that CDH1 potentially plays a vital role in lenvatinib resistance in ICC (**Figures 5B, H**).

## Discussion

The PDX model has become a prevalent platform for pre-clinical drug screening due to the ability to recapitulate tumor biology. Moreover, this tool has several advantages when compared to the use of conventional cells lines (30). Therefore, in the era of precision medicine, PDX models have become a valuable tool. In this study, we successfully established PDX models derived from 49 patients with ICC, with an engraftment rate of 55.1%. PDX establishment was associated with negative HBsAg, the presence of mVI, poorer tumor differentiation, multiple tumor number, presence of lymph node metastasis and later TNM stage. Patients with PDX establishment had a higher propensity to relapse and a lower survival rate after surgery. Furthermore, the established PDX models can be used to predict treatment response and to identify potential mechanisms of drug resistance. Therefore, the PDX platform will contribute to advancing precision medicine for the management of ICC.

We characterized the preserved histological and genetic features of PDXs. The results demonstrated that histological morphology and ICC markers (CK7 and CK19) were well maintained during passages. The consistency of these markers was important for evaluating the utility of selected therapeutic drugs, which represent the pathological similarity between PDXs and primary tumors (31). Noticeably, Ki 67 expression was relatively low in the PDX F2 passage, and Ki67 expression increased in following F4 and F6 passages. This may account for the phenomenon that the latency period becomes shorter during the following PDX passages (32, 33). Additionally, expression of human-derived CD34 gradually decreased, implying that human stromal elements can change as the engrafted tumors settle into their new tumor microenvironment (34). The speed and extent of the drift within stromal components remains elusive, and the impact of drug tests remain controversial. Therefore, PDX models within 10 passages are considered appropriate for drug testing in light of the preservation of parental tumor histopathological and genetic information (35, 36). Our examination of gene expression and SNP profiles revealed that the PDX models could maintain the heterogeneity of the primary tumor and the homogeneity of the patients during passage, which is consistent with results previously reported (30, 36, 37).

In this study, we found that presence of mVI, poorer tumor differentiation, multiple tumor number, presence of lymph node metastasis, and later TNM stage were associated with higher PDX establishment rate. These factors are also indicative of aggressive biological characteristics and poor outcome (5). Moreover, successful engraftment indicated reduced TTR and OS. Therefore, PDX formation from resected tumor specimens without excessive *in vitro* manipulation reflects the authentic tumor biology of the most aggressive tumors (34). To the best of our knowledge, this is the first study to evaluate the relationship between tumor engraftment in PDX models and clinical outcomes in ICC patients (18). PDX establishment may help identify the most malignant tumors and treatment strategies based on the evaluation of drug sensitivity in PDX models could be used to improve patient clinical outcomes.

The prerequisite step for downstream application and analysis is to obtain stable and high take rates for facilitating the broad application of PDX models. Several technical aspects of PDX establishment should be considered. In this study, tissue mince was incubated in growth factors (basic fibroblast growth factors and human epidermal growth factor) and then incubated with Matrigel™. A transplant needle was used to implant these tissues into the subcutaneous area of right flanks. We obtained a relative high take rate of 55.1%. The addition of growth factors and collagen may promote the survival of tumor cells (38, 39). Additionally, the use of transplant needles simplified implantation procedures, and reduced the failure rate caused by potential infection (40). Furthermore, we also managed to establish PDX models derived from metastatic lesions. The most commonly used tissues derived from surgical specimens and other more convenient approaches, including fine-needle aspiration and endoscopic biopsy, should be investigated to establish PDXs to expand their practical application. Recent literature reports show that PDXs have been established using gastroscopic biopsies in gastric cancer, and fine needle aspirate in primary pancreatic ductal adenocarcinoma and metastatic sites, which largely promote application of PDX engraftment in various resectable and unresectable malignancies (32, 41).

Lenvatinib has been approved as first-line treatment for advanced hepatocellular carcinoma in the US, China, Japan, and many other countries (42). As a counterpart of hepatocellular carcinoma, treatment efficacy of lenvatinib plus PD-1 antibody has been observed in a subpopulation of patients with ICC (43). In this study, gene expression and SNP data based on the lenvatinib-sensitive and resistant PDX models indicate that CDH1 is likely to regulate lenvatinib resistance. However, the specific mechanism through which this occurs requires further study. Because PDX models authentically reflect the histological and genomic characteristics of original tumors, they may represent a powerful modality to replicate patients’ treatment response and clinical efficiency. One large-scale study that included tumor samples from 1163 patients with a variety of advanced solid tumors revealed a sensitivity of 96% and specificity of 70% in drug screening (44). These results indicate that PDX models are capable of guiding oncologists to the most efficient treatments (44). For example, patients IMF-138 and IMF-114 achieved satisfactory treatment efficiency following the drug screening results of PDX models. As a functional diagnosis platform, PDX can produce convincing results when next generation sequencing results are unclear or controversial with respect to drug administration, emphasizing the role of functional diagnostic models (45).

PDX models provide an opportunity for preclinical drug-screening and personalized cancer treatment. Several obstacles should be overcome before PDX models are a routine part of clinical practices. One major challenge is time, with the average time between engraftment and drug exposure across various implanted tumors being 16 weeks. During this period, some patients may lose the opportunity to use the resultant treatment regimens, which undermines the practical value of this technique in clinical decision-making. Therefore, PDX- or primary tumor-based rapid drug screening assays should be developed (44, 46). Another challenge is that engraftment failure is still high for ICC. A take rate of at least 60%–70% is required for personalized medicine strategies, and this aspect of PDX development requires further improvement (8).

In summary, we have developed a panel of PDXs from patients with ICC that authentically recapitulate the parental tumors and demonstrate that PDX establishment can function as an independent indicator of clinical outcomes. Additionally, *in vitro* patient derived cell line of patient derived organoid drug screening followed by *in vivo* PDX validation will provide a practical framework for cancer drug discovery. Although some factors still hinder widespread clinical application, including the long timeframe for engraftment and lack of immune components, extensive research will eventually facilitate the implementation of PDXs in personalized medicine and to improve survival rates in patients with cancer.

## Data Availability Statement

The datasets presented in this study can be found in online repositories. The names of the repository/repositories and accession number(s) can be found below: NCBI and accession number PRJNA729191 (https://www.ncbi.nlm.nih.gov/sra/PRJNA729191).

## Ethics Statement

The studies involving human participants were reviewed and approved by Ethics Board at the Liver Cancer Institute of Zhongshan hospital, Fudan University. The patients/participants provided their written informed consent to participate in this study. The animal study was reviewed and approved by Animal Care and Use Committee of Zhongshan Hospital of Fudan University.

## Author Contributions

YG, RZ, J-FH, and H-XP performed the experiments and analyzed the data. BH, P-XW, and RZ contributed reagents and materials. YG, X-RY, J-WC, and X-WH conceived and designed the experiments. WG, JZ, and JF discussed the manuscript. JF and X-RY critically reviewed the manuscript. YG contributed to drafting and revising the manuscript. All authors contributed to the article and approved the submitted version.

## Funding

This study was jointly supported by the National Key R&D Program of China (2019YFC1315800, 2019YFC1315802), the State Key Program of National Natural Science of China (81830102), National Natural Science Foundation of China (81772578, 81772551, 81872355 and 82072715), the Shanghai Municipal Health Commission Collaborative Innovation Cluster Project (2019CXJQ02), Shanghai “Rising Stars of Medical Talent” Youth Development Program (Outstanding Youth Medical Talents), Zhong-shan Hospital Science Foundation (2020ZSQN87), the Projects from the Shanghai Science and Technology Commission (19441905000), Shanghai Municipal Key Clinical Specialty.

## Conflict of Interest

Author H-XP was employed by companies Shanghai Dunwill Medical Technology Co., Ltd and Shanghai Epione Medlab Co., Ltd.

The remaining authors declare that the research was conducted in the absence of any commercial or financial relationships that could be construed as a potential conflict of interest.
